# Relationships between Quadriceps Tendon Elasticity and Knee Flexion Angle in Young Healthy Adults

**DOI:** 10.3390/medicina55020053

**Published:** 2019-02-15

**Authors:** Bungo Ebihara, Hirotaka Mutsuzaki, Takashi Fukaya

**Affiliations:** 1 Graduate School of Health Sciences, Ibaraki Prefectural University of Health Sciences, 4669-2 Ami, Ami-machi, Inashiki-gun, Ibaraki 300-0394, Japan; 2 Department of Rehabilitation, Tsuchiura Kyodo General Hospital, 4-1-1 Otsuno, Tsuchiura, Ibaraki 300-0028, Japan; 3 Department of Orthopaedic Surgery, Ibaraki Prefectural University of Health Sciences, 4669-2 Ami, Ami-machi, Inashiki-gun, Ibaraki 300-0394, Japan; mutsuzaki@ipu.ac.jp; 4 Department of Physical Therapy, Faculty of Health Sciences, Tsukuba International University, 6-8-33 Manabe, Tsuchiura, Ibaraki 300-0051, Japan; t-fukaya@tius.ac.jp

**Keywords:** quadriceps tendon, elastography, knee flexion angle, sex, leg dominance

## Abstract

*Background and objectives:* Although tendon elasticity by elastography is useful for diagnosing tendon disorders and planning rehabilitation regimens of the tendon, there are few reports on the quadriceps tendon. Moreover, relationships between the quadriceps tendon elasticity and knee angle have not been investigated. The purpose of this study was to clarify the relationship between quadriceps tendon elasticity and knee flexion angle in young healthy adults using elastography, and to investigate the difference in elasticity by sex and leg dominance. *Materials and Methods:* A total of 40 knees in 20 young healthy adults were included in this study (age: 25.5 (23.3–27.5) years). At knee flexion of 30°, 60°, and 90°, quadriceps tendon elasticity was measured using ShearWave™ Elastography during the ultrasound examination. *Results:* There were significant differences in the elasticity between all angles (*p* < 0.001). Elasticity was increased more at 60° than at 30° and at 90° than at 60°. Elasticity in men was higher than that in women at 60° (*p* = 0.029). There were no differences (*p* = 0.798) in elasticity at each angle between the dominant and non-dominant legs. *Conclusions:* The quadriceps tendon elasticity increased according to the knee flexion angle in young healthy adults. Moreover, elasticity was affected by sex, but not by leg dominance. Clinically, in a rehabilitation regimen, attention should be paid to exercises that could increase stiffness accompanying flexion of the knee to avoid further tendon damage as risk management in the acute phase.

## 1. Introduction

Elastography is a term referring to imaging techniques that aim to assess tissue stiffness, such as the elastic value. Although elastography has been widely used for the diagnosis of breast cancer, liver fibrosis, and thyroid nodules, its musculoskeletal applications are beginning to be realized [1]. There are several types of elastography. ShearWave™ Elastography (SWE™) (Supersonic Imaging, Aix-en-Provence, (Provence-Alpes-Côte d’Azur), France) is one type of elastography that uses an ultrasound unit. Tissue stiffness is generally measured by Young’s modulus. SWE™ generates shear waves in the body and calculates Young’s modulus from their propagation speed [2]. The reliability of SWE™ has been verified [3,4]. SWE™ has been used for musculoskeletal disorders [5,6,7].

Musculoskeletal elasticity is one of the mechanical properties of tissues, which is affected by age [8,9], body mass index (BMI) [10,11], sex and muscle strength [11], and many neuromuscular and orthopedic disorders [12]. In the assessment of tendon pathology, elastography is expected to provide an early diagnosis, to identify the risk of injury, and to support the evaluation of rehabilitation interventions [13]. However, there is a lack of consensus in the application of elastography to detect tendon pathology [14].

Quadriceps tendon disorders include tendon ruptures [15], anterior knee pain after anterior cruciate ligament (ACL) reconstructions using quadriceps tendon–patellar bone autograft [16], and jumper’s knee, defined as an insertional tendinopathy [17]. Elastography may be useful for early diagnosis, identifying risk of injury, and supporting the evaluation of rehabilitation interventions of these conditions [13]. To compare the normal and abnormal elasticity of the quadriceps tendon, measurement of normal elastic values is necessary as a basic study. However, few studies have investigated quadriceps tendon elasticity by elastography [14].

Several elastic properties of patellar tendons have been reported by previous studies employing elastography. Patellar tendon stiffness was higher in men than in women [11]. In addition, there is no difference in patellar tendon elasticity between the dominant and non-dominant legs [18]. Focusing on the relationship between tendon elasticity and joint movement, it is reported that patellar tendon elasticity increased with knee flexion [19,20]. Patellar tendons categorized as soft in volleyball players had lower Victorian Institute of Sport Assessment–Patella scores than those categorized as hard [21]. Patellar tendon strain ratios of the knees that underwent ACL reconstruction using bone–tendon–bone autograft were lower than those of the healthier sides [22].

The purpose of this study was to clarify the normal elastic values of the quadriceps tendon. Therefore, we investigated the relationship between quadriceps tendon elasticity and knee flexion angle in young healthy adults using SWE™. In addition, we clarified the difference in elasticity by sex and leg dominance. The quadriceps tendon and patellar tendon are connected via the patella. Moreover, superficial fibers of the quadriceps tendon become continuous with the patellar tendon [23]. From the abovementioned data, we predict that the quadriceps tendon has elastic properties similar to the patellar tendon. Our first hypothesis was that the quadriceps tendon elasticity increases when the knee is flexed. Second, men’s quadriceps tendons are stiffer than women’s quadriceps tendons. Third, there is no difference in elasticity between the dominant and non-dominant legs.

## 2. Materials and Methods

### 2.1. Participants

This study was conducted in 2018. A total of 40 knees in 20 young healthy adults were evaluated in this study. These participants were not athletes. The inclusion criteria were as follows: participants who could follow instructions during measurement and had no fever, joint pain, and muscle pain. Moreover, there were no physical findings indicating dehydration, such as dry mouth and skin. The exclusion criteria were a history of knee injuries and diseases. We obtained data of the participants’ age, sex, height, weight, BMI, and dominant leg. Dominant leg was defined as the leg they would use to kick a ball [24].

### 2.2. Ethics Statement

The ethics committee of Ibaraki Prefectural University of Health Sciences and Tsuchiura Kyodo General Hospital approved this study (Nos. e159 and 690, respectively). Written informed consent was obtained from each participant.

### 2.3. Measurement of Knee Range of Motion (ROM)

Participants were assessed while they were in a supine position on the bed. We measured knee range of extension and flexion. The ROM was measured through passive and active movements. Knee ROM was measured using goniometry, with a minimum value of 1°. The landmarks used in the measurements were the greater trochanter of the femur, lateral condyle of the femur, fibular head, and the lateral malleolus of the fibula.

### 2.4. Measurement of Knee Extension Strength

Participants were assessed while they were in a sitting position. The maximal isometric knee extension was measured with the Biodex System 3C dynamometer (Biodex Medical Systems, NY, USA) at 70° knee flexion [25]. The angle of the backrest of the seat was set to 85°. The waist, lower trunk, and thigh were fixed to the seat with straps. The strap at the distal end of the lever arm of the dynamometer was tied to the lower leg directly above the medial malleolus. Participants performed 5 s of maximal voluntary isometric contraction and were verbally encouraged to reach maximal effort [18]. The peak torque was recorded, with a minimum value of 1 Nm. Moreover, according to the report by Kigawa et al. [25], the weight-bearing index (WBI) was calculated.

### 2.5. Measurement of Quadriceps Tendon Elasticity

Ultrasound examinations were performed with the Aixplore^®^ ultrasound unit in conjunction with a 2- to 10-MHz liner transducer (Supersonic Imaging, Aix-en-Provence, France). A preset of musculoskeletal and knee was selected. Quadriceps tendon elasticity was measured by SWE™ of the Aixplore^®^. SWE™ provides the value of elasticity in kPa, with a minimum value of 1 kPa. The limit of measurement was 800 kPa. The room temperature was controlled at 25 °C [26]. All the ultrasound examinations were performed by the same physical therapist with 5 years’ experience in performing musculoskeletal ultrasound examinations.

Participants were assessed under supine positions with 30°, 60°, and 90° of knee flexion. Measurement at the extension position was not carried out to avoid anisotropy due to the quadriceps tendon concave status [27]. Instead, 30° of knee flexion was chosen. In addition, since the value of elasticity exceeded the limit of measurement, it was impossible to measure at 120° of knee flexion. Therefore, 30°, 60°, and 90° of knee flexion were selected. With each knee flexion angle, towels and cushions were placed under the knee to relax it. During the examinations, verbal instruction was given to the participants to stay relaxed and avoid any muscle contraction. After taking the posture measurements, first, the muscle tendon transition part of rectus femoris muscle was identified and it was marked with a gel on the skin, using the B-mode horizontal axis image. Second, the center of the base of the patella was identified and it was marked with a gel by palpation on the skin. Finally, a large amount of gel was used, and the transducer was placed on the straight line of the two marks with the lightest transducer pressure [26]. Based on the study that considered the validity of elastography in skeletal muscle [28], quadriceps tendon fibers and the transducer were made parallel while watching the B-mode image. Then, SWE™ was activated. SWE™ Opt was set to penetration mode. The transducer was kept motionless for 5 to 10 s during the acquisition of the SWE™ sonogram video.

**Figure 1:**
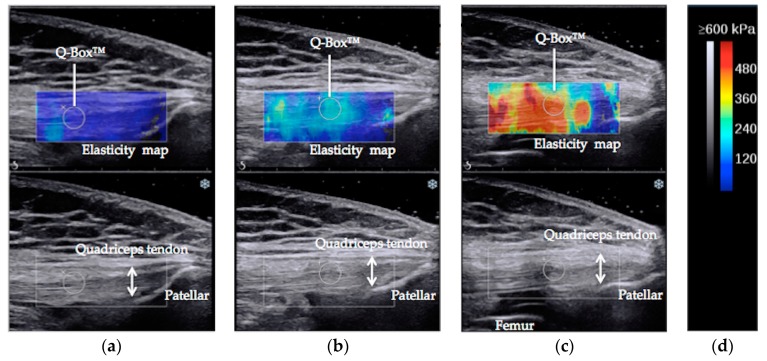
Typical example of elasticity maps of quadriceps tendon during passive knee flexion at 30° (**a**), 60° (**b**), and 90° (**c**). The gray and color scale next to the elasticity maps (**d**).

The SWE™ sonogram video was converted to a still image when the elasticity map of the quadriceps tendon was stable. For the elasticity measurement, Q-box™ of the internal function of the Aixplorer^®^ was used. The Q-box™ was placed 2 cm proximal to the bony insertion onto the patella (Figure 1) [4]. A circle delineating the Q-box™ was set as large as possible within the quadriceps tendon. Average elastic value was recorded for statistical analysis [26].

The same measurements were repeated 15 min after the first measurements for verification of the reliability of the measurements. The intraclass correlation coefficient (ICC) was calculated from the average value of elasticity of the first and second times. The ICC (1.1) values of the quadriceps tendon elasticity at 30°, 60°, and 90° of knee flexion were 0.906, 0.930, and 0.803, respectively. The ICC values above 0.75 are indicative of good reliability, and those below 0.75 indicate poor-to-moderate reliability [29].

### 2.6. Statistical Analysis

Elasticity data of the quadriceps tendon were analyzed in a three-way (2 × 2 × 3) repeated measures analysis of variance (ANOVA) with sex (men and women), leg dominance (dominant and non-dominant), and knee flexion angle (30°, 60°, and 90°) as factors. However, the interactions between sex and flexion angle were recognized. For that reason, we added a one-way repeated measures ANOVA using the elasticity data of the dominant legs with knee flexion angle (30°, 60°, and 90°) as factors separately for men and women. Furthermore, when the main effect was observed, Bonferroni post hoc testing was performed.

Distribution of data was judged by the Shapiro–Wilk test. A parametric test was selected for data of the normal distribution; otherwise a nonparametric test was selected.

To evaluate the differences between men and women, two sample t-tests were conducted in height, weight, BMI, active extension, passive extension, extension peak torque, WBI, elasticity of 30°, and elasticity of 60°. The Mann–Whitney test was conducted in age, active flexion, passive flexion, and elasticity of 90°.

In order to evaluate the differences between the dominant and non-dominant legs, a paired t-test was conducted in active extension, passive extension, extension peak torque, and WBI. A Wilcoxon signed–rank test was conducted in active flexion and passive flexion.

The *p* values <0.05 were considered to be statistically significant. SPSS^®^ statistics version 24.0 (IBM, NY, USA) was used to perform all the statistical analyses.

## 3. Results

### 3.1. The Participant Characteristics

The participant characteristics, including age, sex, height, weight, BMI, and dominant leg are summarized in Table 1.

### 3.2. The Results Between Men and Women

**Figure 2:**
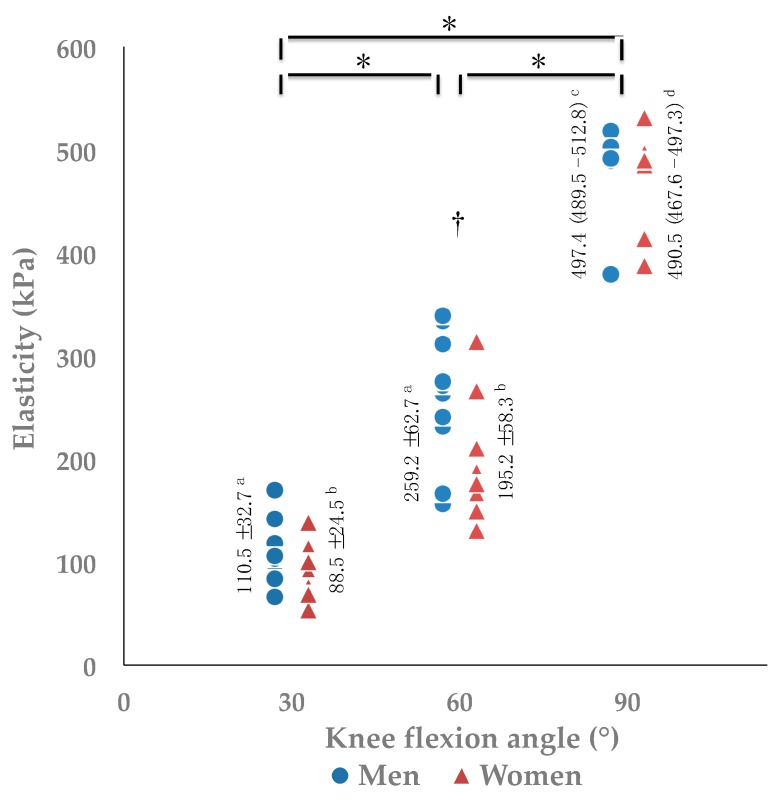
Comparisons of elasticity between men and women at 30°, 60°, and 90° of knee flexion. * One-way repeated measures ANOVA and Bonferroni post hoc testing detected significant difference among all angles (*p* < 0.001). ^†^ Two sample *t*-tests detected significant difference between sexes (*p* = 0.029). ^a^ Elasticity values of men are represented as mean ±SD. ^b^ Elasticity values of women are represented as mean ± SD. ^c^ Elasticity values of men are represented as median (interquartile range). ^d^ Elasticity values of women are represented as median (interquartile range).

The results between men and women are summarized in Figure 2 and Table 2. There are significant main effects for angle in both men (*F*(2, 18) = 377.149, *p* < 0.001, *pη*^2^ = 0.977, *power* = 1.000) and women (*F*(2, 18) = 255.001, *p* < 0.001, *pη*^2^ = 0.966, *power* = 1.000) (Figure 2). Post hoc testing detected significant differences on the elasticity of quadriceps tendons among all angles (*p* < 0.001), and the values of elasticity increased as the knee flexed (Figure 2). Elasticity of men was higher than that of women at 60° (*p* = 0.029), but not at 30° (*p* = 0.105) and 90° flexion (*p* = 0.247). The height and extension peak torque of men was greater than those of women (*p* < 0.05) (Table 2). Knee passive flexion ROM was greater in women than that in men (*p* = 0.002) (Table 2).

### 3.3. The Results between the Dominant and Non-Dominant Legs

**Figure 3:**
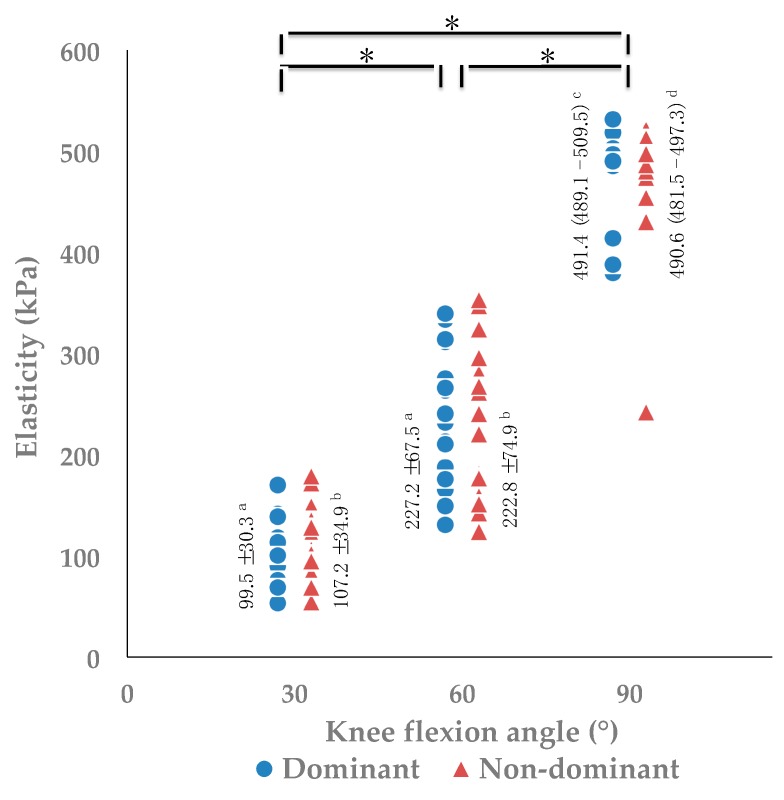
Comparisons of elasticity between the dominant and non-dominant legs at 30°, 60°, and 90° of knee flexion. * Three-way repeated measures ANOVA and Bonferroni post hoc testing detected significant difference among all angles (*p* < 0.001). ^a^ Elasticity values of the dominant leg are represented as mean ± SD. ^b^ Elasticity values of the non-dominant leg are represented as mean ± SD. ^c^ Elasticity values of the dominant leg are represented as median (interquartile range). ^d^ Elasticity values of the non-dominant leg are represented as median (interquartile range).

The results between the dominant and non-dominant legs are summarized in Figure 3 and Table 3. There are significant main effects for angle (*F*(2, 36) = 641.295, *p* < 0.001, *pη*^2^ = 0.973, *power* = 1.000), but none for leg dominance (*F*(1, 18) = 0.067, *p* = 0.798, *pη*^2^ = 0.004, *power* = 0.057) (Figure 3). Post hoc testing detected significant differences on the elasticity of quadriceps tendon among all three angles (*p* < 0.001), and the values of elasticity increased as the knee flexed (Figure 3). Knee active flexion ROM was greater in the non-dominant leg than in the dominant leg (*p* = 0.027) (Table 3).

## 4. Discussion

This study clarified the relationship between quadriceps tendon elasticity and knee flexion angle in young healthy adults using elastography. The values of elasticity increased as the knee flexed. Moreover, men’s quadriceps tendons are stiffer than women’s quadriceps tendons at knee flexion of 60°. However, there is no difference between the dominant and non-dominant legs.

Quadriceps tendon elasticity increased as the knee flexed in this study. In a previous report that studied muscles, elasticity and passive force had a highly linear relationship [30]. When the knee flexion angle exceeds approximately 40°, the rectus femoris, vastus medialis oblique, and vastus lateralis muscle start to generate passive tension [31]. This passive tension is transmitted to the quadriceps tendon. Moreover, passive tension is generated to the patellar tendon with knee flexion. It is clarified that elasticity of the patellar tendon increases with knee flexion [19,20]. This passive tension is also transmitted to the quadriceps tendon. These passive tensions from the quadriceps muscle and patellar tendon increased the quadriceps tendon elasticity.

Men’s quadriceps tendons were stiffer than women’s quadriceps tendons at a knee flexion of 60°. The quadriceps muscle strength of men was higher than that of women in this study. In a previous study, there was a correlation between knee extension moment and quadriceps muscle volume [32]. From this previous study, men’s quadriceps muscle volume may be larger than that of women. Muscle elasticity is associated with passive joint stiffness in the joint position where the muscle is sufficiently lengthened [33]. Muscle volume is a good predictor of angular stiffness [34]. Because the muscle volume was larger in men, the men’s quadriceps muscles may have generated larger passive tension than those of the women’s when the knee flexion angle exceeded approximately 40°. Therefore, that may be why the tendon became stiff at 60° in men. Moreover, regarding the reason why men’s tendons are stiffer than women’s, the differences in synthesis of collagen due to weight, muscle strength, and hormone influence can be considered [11]. In this study, muscle strength was higher in men. With this influence, the quadriceps tendons may become stiff in men.

There was no difference of elasticity between the dominant and non-dominant legs. There was no difference in quadriceps tendon elasticity between the dominant and non-dominant legs of athletes [27]. The result of this study was the same as that of the previous study. Because it was the same person and there was no difference in muscle strength between the dominant and non-dominant legs, it may be that there was no difference in quadriceps muscle volume and synthesis of collagen. Therefore, there may have been no difference in the quadriceps tendon elasticity.

Moreover, there was no difference in elasticity between men and women at 90° of knee flexion. In all positions of flexion beyond 90°, the patellar facets of the femur demonstrate an extensive contact area with the broad posterior surface of the quadriceps tendon [35]. From this report, it is thought that compression of the tendon increases elasticity. We thought that the difference in elasticity between men and women disappeared because the compressive force exceeded the influence of the leg muscle mass. Moreover, in a previous report that studied quadriceps tendon and femoral pressure [36], the average tendofemoral pressure was 1.6 ± 0.3 MPa at 120° of knee flexion. Given this high pressure, it was impossible to measure elasticity at 120° of knee flexion in this study.

Clinically, in a rehabilitation regimen, attention should be paid to exercises that could increase stiffness with accompanying flexion of the knee in order to avoid further tendon damage as risk management in the acute phase. Elastography detects alterations of tendinopathy at an early stage [37], and symptomatic tendons exhibit significantly lower mean SWE™ values than healthy tendons [38]. The data of this study may be useful for early diagnosis of quadriceps tendon ruptures, anterior knee pain, and jumper’s knee, because the relationship between angle and elasticity was clarified. In detecting abnormality, it is necessary to unify the flexion angle of the knee to prevent increases in elasticity and decreases in accompanying angle change. Moreover, if quadriceps muscle contraction is present, tension is transmitted to the quadriceps tendon and the values of elasticity increase, so measurement for diagnosis should be done when the subject is as relaxed as possible. Furthermore, SWE™ values were closely correlated to patient’s clinical symptoms [38]. Elastography may be used as a tool to evaluate whether an exercise regimen is appropriate for the patient to prevent further tendon damage [37]. That is, when damage is applied to the tendon, the symptoms worsen, and the values of elasticity will decrease. Therefore, it is also important to capture the changes of elasticity over time. It would be better to adjust to an exercise load that does not decrease the elastic values in tendinopathy.

There are limitations in this study. First, the participants were all young. Therefore, it is necessary to conduct related studies in the future involving participants with varying ages. Second, myoelectrical activity was not recorded to ensure that the muscles were relaxing. Combined use of electromyography is necessary for more reliable verification. Finally, the quadriceps muscle volume was not measured. Therefore, there is ambiguity in the relationship between quadriceps muscle volume and quadriceps tendon elasticity. Further studies are needed regarding these points.

## 5. Conclusions

The quadriceps tendon elasticity increases along with knee flexion in young healthy adults. Elasticity is higher in men than in women at a knee flexion of 60°, and elasticity is not affected by leg dominance. In a rehabilitation regimen, attention should be paid during exercises that could increase stiffness when accompanied by flexion of the knee in order to avoid further tendon damage as risk management in the acute phase.

## Figures and Tables

**Table 1:** Participant characteristics.

Parameters	*n* = 20
Age (years)	25.5 (23.3–27.5) ^1^
Sex (men/women)	10/10
Height (cm)	166.2 ± 6.0 ^2^
Weight (kg)	59.7 ± 6.6 ^2^
BMI (kg/m^2^)	21.6 ± 2.2 ^2^
Dominant leg (right/left)	19/1

^1^ Values are represented as median (interquartile range). ^2^ Values are represented as mean ± standard deviation (SD). Abbreviation: BMI = body mass index.

**Table 2:** Comparison of characteristics and measured values between men and women.

Parameters	Men (*n* = 10)	Women (*n* = 10)	*p*-Value
Age (years)	24.5 (23.0–26.8)	26.0 (24.0–28.3)	0.481 ^a^
Height (cm)	170.5 ± 3.6	161.9 ± 4.7	<0.001 *^,b^
Weight (kg)	62.0 ± 6.6	57.3 ± 6.1	0.115 ^b^
BMI (kg/m^2^)	21.3 ± 1.9	21.9 ± 2.6	0.564 ^b^
Active extension (°)	7.1 ± 3.2	5.9 ± 2.1	0.331 ^b^
Active flexion (°)	146.0 (142.8–147.0)	145.0 (143.8–146.3)	0.853 ^a^
Passive extension (°)	7.1 ± 3.2	5.9 ± 2.1	0.331 ^b^
Passive flexion (°)	154.0 (151.8–155.3)	157.0 (155.8–157.0)	0.002 *^,a^
Extension peak torque (Nm)	189.9 ± 28.2	150.2 ± 34.1	0.011 *^,b^
WBI	1.04 ± 0.16	0.91 ± 0.19	0.125 ^b^

Characteristics and measured values are represented as median (interquartile range) or mean ±SD. * *p* < 0.05. ^a^ Mann-Whitney test. ^b^ Two sample *t*-test. Abbreviations: BMI = body mass index; WBI = weight bearing index.

**Table 3:** Comparison of measured values between the dominant and non-dominant legs.

Parameters	Dominant (*n* = 20)	Non-Dominant (*n* = 20)	*p*-Value
Active extension (°)	6.5 ± 2.7	6.5 ± 2.6	1.000 ^b^
Active flexion (°)	145.0 (143.3–146.8)	146.0 (145.0–146.8)	0.027 *^,a^
Passive extension (°)	6.5 ± 2.7	6.5 ± 2.6	1.000 ^b^
Passive flexion (°)	155.5 (153.3–157.0)	155.5 (153.5–156.0)	0.317 ^a^
Extension peak torque (Nm)	170.1 ± 36.6	168.4 ± 44.2	0.744 ^b^
WBI	0.98 ± 0.18	0.97 ± 0.22	0.752 ^b^

Measured values are represented as median (interquartile range) or mean ±SD. *p < 0.05. ^a^ Wilcoxon signed–rank test. ^b^ Paired *t*-test. Abbreviation: WBI = weight bearing index.
